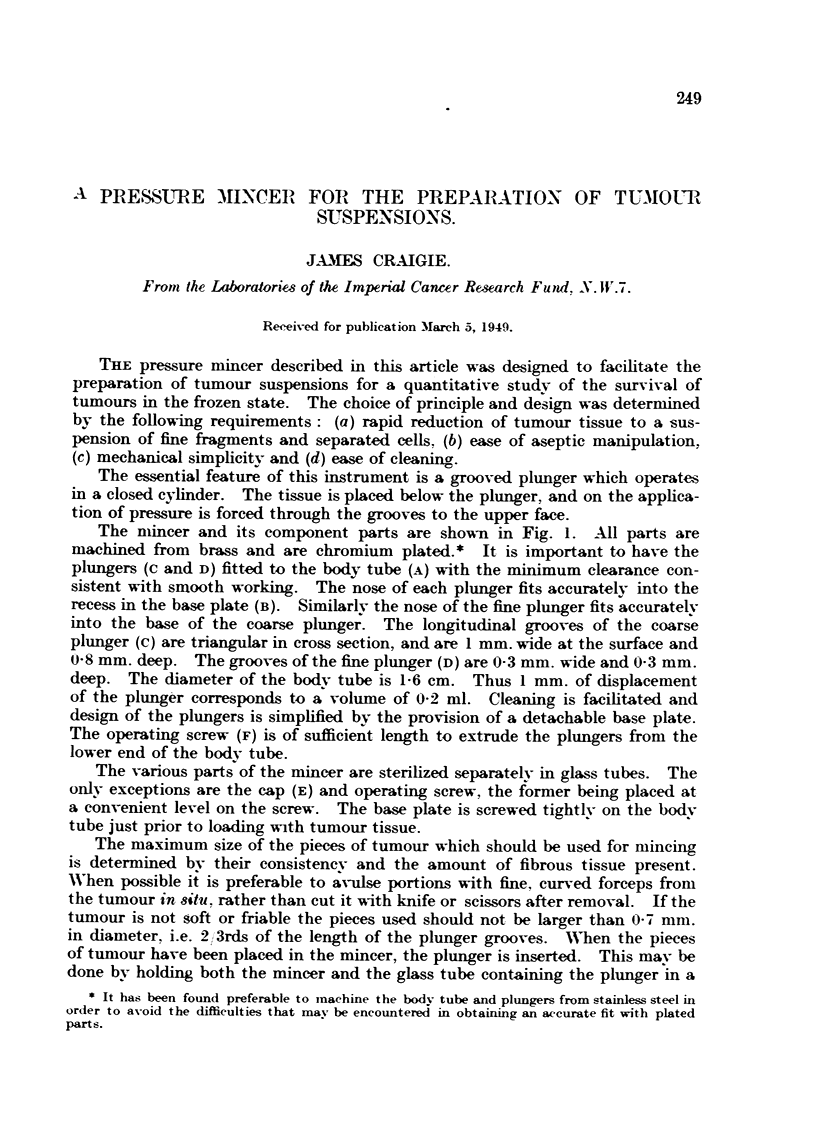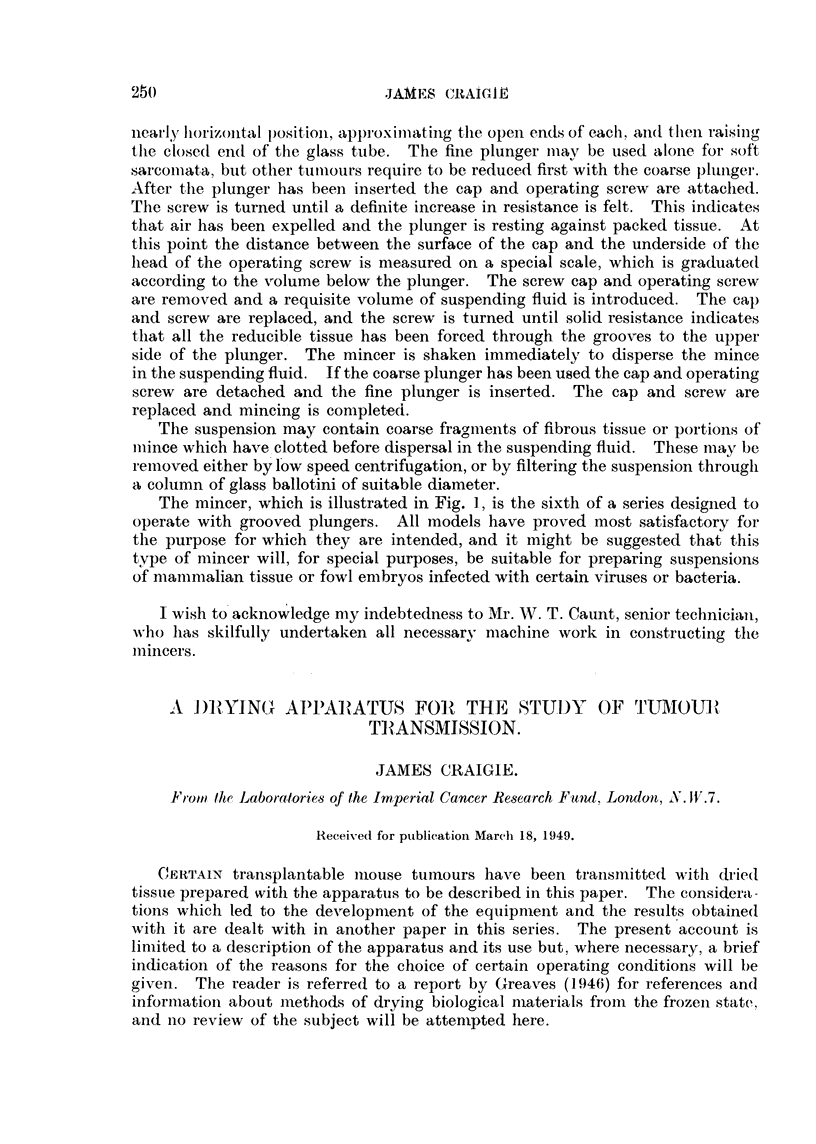# A Pressure Mincer for the Preparation of Tumour Suspensions

**DOI:** 10.1038/bjc.1949.26

**Published:** 1949-06

**Authors:** James Craigie


### A PRESSURE MINCER FOR THE PREPARATION OF TUMOUR SUSPENSIONS.

JAMES CRAIGIE.

*From the Laboratories of the Imperial Cancer Research Fund, N.W.7.*

Received for publication March 5, 1949.

THE pressure mincer described in this article was designed to facilitate the preparation of tumour suspensions for a quantitative study of the survival of tumours in the frozen state. The choice of principle and design was determined by the following requirements : (*a*) rapid reduction of tumour tissue to a suspension of fine fragments and separated cells, (*b*) ease of aseptic manipulation, (*c*) mechanical simplicity and (*d*) ease of cleaning.

The essential feature of this instrument is a grooved plunger which operates in a closed cylinder. The tissue is placed below the plunger, and on the application of pressure is forced through the grooves to the upper face.

The mincer and its component parts are shown in Fig. 1. All parts are machined from brass and are chromium plated.* It is important to have the plungers (C and D) fitted to the body tube (A) with the minimum clearance consistent with smooth working. The nose of each plunger fits accurately into the recess in the base plate (B). Similarly the nose of the fine plunger fits accurately into the base of the coarse plunger. The longitudinal grooves of the coarse plunger (C) are triangular in cross section, and are 1 mm. wide at the surface and 0·8 mm. deep. The grooves of the fine plunger (D) are 0·3 mm. wide and 0·3 mm. deep. The diameter of the body tube is 1·6 cm. Thus 1 mm. of displacement of the plunger corresponds to a volume of 0·2 ml. Cleaning is facilitated and design of the plungers is simplified by the provision of a detachable base plate. The operating screw (F) is of sufficient length to extrude the plungers from the lower end of the body tube.

The various parts of the mincer are sterilized separately in glass tubes. The only exceptions are the cap (E) and operating screw, the former being placed at a convenient level on the screw. The base plate is screwed tightly on the body tube just prior to loading with tumour tissue.

The maximum size of the pieces of tumour which should be used for mincing is determined by their consistency and the amount of fibrous tissue present. When possible it is preferable to avulse portions with fine, curved forceps from the tumour *in situ*, rather than cut it with knife or scissors after removal. If the tumour is not soft or friable the pieces used should not be larger than 0·7 mm. in diameter, i.e. 2/3rds of the length of the plunger grooves. When the pieces of tumour have been placed in the mincer, the plunger is inserted. This may be done by holding both the mincer and the glass tube containing the plunger in a

* It has been found preferable to machine the body tube and plungers from stainless steel in order to avoid the difficulties that may be encountered in obtaining an accurate fit with plated parts.

nearly horizontal position, approximating the open ends of each, and then raising the closed end of the glass tube. The fine plunger may be used alone for soft sarcomata, but other tumours require to be reduced first with the coarse plunger. After the plunger has been inserted the cap and operating screw are attached. The screw is turned until a definite increase in resistance is felt. This indicates that air has been expelled and the plunger is resting against packed tissue. At this point the distance between the surface of the cap and the underside of the head of the operating screw is measured on a special scale, which is graduated according to the volume below the plunger. The screw cap and operating screw are removed and a requisite volume of suspending fluid is introduced. The cap and screw are replaced, and the screw is turned until solid resistance indicates that all the reducible tissue has been forced through the grooves to the upper side of the plunger. The mincer is shaken immediately to disperse the mince in the suspending fluid. If the coarse plunger has been used the cap and operating screw are detached and the fine plunger is inserted. The cap and screw are replaced and mincing is completed.

The suspension may contain coarse fragments of fibrous tissue or portions of mince which have clotted before dispersal in the suspending fluid. These may be removed either by low speed centrifugation, or by filtering the suspension through a column of glass ballotini of suitable diameter.

The mincer, which is illustrated in Fig. 1, is the sixth of a series designed to operate with grooved plungers. All models have proved most satisfactory for the purpose for which they are intended, and it might be suggested that this type of mincer will, for special purposes, be suitable for preparing suspensions of mammalian tissue or fowl embryos infected with certain viruses or bacteria.

I wish to acknowledge my indebtedness to Mr. W. T. Caunt, senior technician, who has skilfully undertaken all necessary machine work in constructing the mincers.

### A DRYING APPARATUS FOR THE STUDY OF TUMOUR TRANSMISSION.

JAMES CRAIGIE.

*From the Laboratories of the Imperial Cancer Research Fund, London, N.W.7.*